# Capturing Cognitive Aging in Vivo: Application of a Neuropsychological Framework for Emerging Digital Tools

**DOI:** 10.2196/38130

**Published:** 2022-09-07

**Authors:** Katherine Hackett, Tania Giovannetti

**Affiliations:** 1 Department of Psychology and Neuroscience Temple University Philadelphia, PA United States

**Keywords:** digital phenotyping, neuropsychology, aging, dementia, smartphone, neurological, psychological, older adults, aging

## Abstract

As the global burden of dementia continues to plague our healthcare systems, efficient, objective, and sensitive tools to detect neurodegenerative disease and capture meaningful changes in everyday cognition are increasingly needed. Emerging digital tools present a promising option to address many drawbacks of current approaches, with contexts of use that include early detection, risk stratification, prognosis, and outcome measurement. However, conceptual models to guide hypotheses and interpretation of results from digital tools are lacking and are needed to sort and organize the large amount of continuous data from a variety of sensors. In this viewpoint, we propose a neuropsychological framework for use alongside a key emerging approach—digital phenotyping. The Variability in Everyday Behavior (VIBE) model is rooted in established trends from the neuropsychology, neurology, rehabilitation psychology, cognitive neuroscience, and computer science literature and links patterns of intraindividual variability, cognitive abilities, and everyday functioning across clinical stages from healthy to dementia. Based on the VIBE model, we present testable hypotheses to guide the design and interpretation of digital phenotyping studies that capture everyday cognition in vivo. We conclude with methodological considerations and future directions regarding the application of the digital phenotyping approach to improve the efficiency, accessibility, accuracy, and ecological validity of cognitive assessment in older adults.

## Introduction

The global burden of dementia, a clinical syndrome associated with cognitive deficits that impair everyday functioning, poses a tremendous and growing challenge to our healthcare system. As the worldwide population of older adults continues to increase and becomes more medically complex and diverse, the number of people that will develop Alzheimer’s disease and related dementias (ADRD) without current pharmacologic treatments to improve cognition and function [1] is expected to triple from 55 million in 2021 to over 139 million by 2050 [2]. Estimates of disability-adjusted life years (ie, sum of years lost due to premature mortality and years lived with disability) indicate that ADRD is extremely burdensome to individuals diagnosed, their families, and their caregivers, ranking among the top 10 most burdensome diseases in the United States [3]. Early diagnosis and intervention before neuronal degeneration and functional disability begin presents one promising route to meaningfully delay disease burden and promote aging in place [4-6]. From a health economics perspective, it is estimated that early detection of the prodromal, mild cognitive impairment (MCI) stage [7] could save $7.9 trillion in the United States alone [8]. Novel digital methods have great potential for efficient, accessible, reliable, and accurate assessment of early cognitive changes reflecting ADRD. However, to be most effective, digital tools should be informed by conceptual models that explain and predict early cognitive changes.

In this viewpoint, we focus on the application of digital phenotyping to assess age-related changes in functional cognition, with contexts of use that include early detection, risk stratification, prognosis, and outcome measurement. We begin by outlining current approaches to detecting pathological cognitive change along with their notable drawbacks. The digital phenotyping approach is introduced as a promising complementary method. We then present a neuropsychological framework of everyday cognitive and functional changes, termed the Variability in Everyday Behavior (VIBE) model, which can be used to inform studies and generate testable hypotheses in the context of digital phenotyping. Supporting literature that was integrated to develop the VIBE model is also summarized. We conclude with methodological considerations and future directions regarding the digital phenotyping approach.

## Current Approaches to Early Detection of Pathological Change

Neurodegenerative pathology may be directly measured in the brain tissue and detected in cerebrospinal fluid (CSF) and blood [9]; biological measures are classified using biomarker-based diagnostic frameworks for ADRD [10,11]. Importantly, existing methods of biomarker testing are expensive, not widely available, and may be invasive depending on the methodology (eg, lumbar puncture). More concerning, however, is that biological indicators of neurodegenerative disease yield limited information on clinical outcomes such as progression, cognitive abilities, and everyday functioning [12,13]. For example, approximately 30% of individuals with substantial amyloid burden—a core Alzheimer’s disease (AD) biomarker—fail to show clinical symptoms of dementia, whereas up to 25% of individuals who meet clinical criteria for AD have no or limited amyloid burden [14]. The prioritization of biological outcomes is also concerning given that clinical outcomes such as cognitive and functional abilities are most predictive of quality of life, cost of care, and independence, which are precisely the outcomes that individuals diagnosed, their caregivers, healthcare professionals, and policy makers most value [15].

Neuropsychological assessment is less expensive and invasive compared to biomarker testing and is currently used for clinical staging, differential diagnosis, tracking change in cognitive functioning over time, and informing personalized recommendations. The neuropsychological measures that are used for clinical assessments have undergone extensive psychometric validation and are informed by cognitive neuroscience theories. At present, neuropsychological test results are a key component of clinical diagnostic criteria for dementia and MCI [10,16,17] and serve as a primary end point in most clinical trials [18]. In recent years, several neuropsychological tests and composite measures have shown promise in identifying very early, subtle changes that occur in presymptomatic disease stages [19-21].

Nevertheless, current assessment methods present methodological drawbacks, including lengthy and resource-intensive in-person testing sessions that are often inaccessible to individuals from underserved or rural communities, highly controlled testing environments that foster limited ecological validity and test anxiety, burdensome and error-prone scoring procedures, and limited data sharing infrastructures. Traditional assessments take place on a single occasion representing a one-time snapshot that may not reflect an individual’s typical range of performance or intervening contextual factors [22,23]. Even when repeat testing is performed, practice effects between sessions may obscure subtle but meaningful cognitive decline [24].

New mobile and computerized platforms with enhanced efficiency and sensitivity, such as repeat ambulatory cognitive assessments, address some of these methodological drawbacks [25] and have been examined in various studies among populations comprising healthy and community-based older adults [22,26], those with preclinical AD [27], and those with MCI or early dementia [28]. However, many of these methods continue to be (A) modeled after traditional tests that measure isolated cognitive domains with limited ecological validity; (B) susceptible to practice effects [29]; (C) influenced by socioeconomic status and cultural factors [30-32]; and (D) prone to challenges with adherence even among highly motivated and engaged individuals, particularly with longer study durations [27,33,34]. Thus, while tremendous advances have been made in the realm of digital cognitive assessment, existing methods continue to show limited generalizability to diverse populations and real-world settings, even when used at home outside of the clinic. The strengths and weaknesses of the current approaches are summarized in Table 1.

**Table 1:** Strengths and weaknesses of current approaches to detect pathological change.

Approaches	Strengths	Weaknesses
Biomarker testing	Objective measurement of disease presence in the body Good sensitivity/early detection of target pathology Ability to localize pathology Ability to identify specific pathology	High cost Limited accessibility Potentially invasive (CSF^a^ and blood biomarkers) Limited correspondence with functional outcomes Limited prognostic value Interpretation can be subjective
Traditional neuropsychological assessment	Extensively validated and informed by cognitive neuroscience theories Noninvasive Measure discrete cognitive abilities Inform personalized recommendations Moderate correspondence with functional outcomes	Limited accessibility Lengthy and error-prone administration and scoring procedures Highly controlled environment and tasks/limited ecological validity Limited sensitivity to early decline Single time point without context Practice effects at repeat administration Influenced by socioeconomic and cultural factors
Mobile cognitive assessment	Brief administration Improved accessibility Potential for increased sensitivity Noninvasive Ability to assess cognition in everyday context and across multiple time points Possible reduction in test anxiety	Challenges in adherence Practice effects at repeat administration Impact of hardware and software differences when personal devices are used Continued impact of socioeconomic/cultural factors Uncontrolled testing environment may lead to increased measurement error/noise

^a^CSF: cerebrospinal fluid.

## Digital Phenotyping

Emerging digital tools lend a unique opportunity to address many of the drawbacks of traditional, computerized, and mobile cognitive testing. One such method is digital phenotyping, an innovative approach that utilizes the “moment-by-moment quantification of the individual-level human phenotype in situ” based on interactions with technology, including smartphones and smart home devices, to capture social and behavioral data passively, continuously, and with minimal interference [35-37]. Because most everyday tasks require the coordinated effort of multiple cognitive processes and are highly context dependent, digital phenotyping data collected in this passive manner may provide a more naturalistic, comprehensive, and nuanced understanding of behavior and cognition as compared to traditional active assessment methods that take place in the clinic, the lab, or during a discrete period of time. Contrary to standardized neuropsychological tasks that are highly related to educational quality [38] and other sociocultural factors [39], digital proxies of everyday behavior captured in someone’s natural environment may yield a less biased measure of cognition and function, particularly when methods rely on longitudinal monitoring of individual change. Furthermore, high frequency continuous data have the potential to improve sensitivity and reliability and reduce the sample size requirements needed to detect subtle differences between groups or among individuals over time [40].

Smartphones, which are ubiquitous, are equipped with a host of embedded sensors that are common across different devices and may be leveraged to passively assess everyday activities and behaviors. Preliminary studies have investigated smartphone-based digital biomarkers (via sensor and application use data) to measure specific behaviors and offer support and validation for call and text message logs [41] as well as call reciprocity [42] as measures of social patterns; WiFi/Bluetooth signals as a proxy for social engagement (time spent proximal to other people) [37]; GPS movement trajectories and keystroke data as measures of mood [43-45]; and accelerometer data to infer sleep patterns [46]. The validity of smartphone digital phenotyping has been demonstrated in mental health and neurological populations, with results supporting the predictive utility of a range of smartphone data for daily stress levels [47], changes in depression and loneliness [43,46,47], psychosis onset and relapse [48-50], suicide risk [47], speech changes [51], and biological rhythms [52].

Other studies have attempted to identify digital markers that reflect underlying cognitive abilities. A 2018 study of 27 healthy young adults [53] followed by a 2019 study of 84 healthy older adults [54] demonstrated significant associations between smartphone metrics (eg, number of apps used, usage by hour of day, swipes, and keystroke events) and performance on standard cognitive tests. Of note, these studies were exploratory in nature and lacked a priori hypotheses to guide analyses. A separate pilot study of adults with and without bipolar disorder examined performance on a digital trail making test and found associations between smartphone typing speed and typing speed variability and test performance, suggesting a possible link between executive functioning and keystroke measures [29]. In the context of MCI and dementia, a feasibility study employing multiple sensor streams and machine learning models identified 5 digital features that discriminated symptomatic (MCI, mild AD) from asymptomatic groups; these features included typing speed, regularity in behavior (via first and last phone use), number of received text messages, reliance on helper apps, and survey compliance [55]. As noted by the authors of the aforementioned pilot and feasibility studies, a major limitation was the small sample sizes, which limited interpretability.

Indeed, although preliminary studies have laid the groundwork for exploring relationships between passive digital variables and standard measures of cognition, the lack of integrative theoretical models to inform interpretation of large continuous datasets represents a major gap [23]. As digital tools and machine learning approaches become increasingly sophisticated, it is critical that theoretically sound models are developed to avoid scattershot analyses and spurious findings and to facilitate interpretability [56]. Furthermore, as technologies inevitably continue to evolve, the development of testable models that are agnostic to hardware and software differences is key to the continued validation of passive approaches [56-58]. Therefore, we propose a neuropsychological framework to guide studies using emerging digital tools to assess age-related cognitive and functional decline. The VIBE model integrates established findings regarding intraindividual variability, cognitive abilities, and everyday functioning in the context of aging and ADRD. Importantly, the VIBE model generates specific, testable hypotheses grounded in theory that may inform the design and interpretation of future digital phenotyping studies and represents a preliminary step toward establishing conceptual guidelines for the field.

## Approach to Framework Development

The VIBE model resulted from an in-depth review of the neuropsychology, neurology, neuroscience, rehabilitation psychology, and computer science literature. Consistent findings in both performance level and intraindividual variability were identified across the spectrum of cognitive impairment and interpreted in the context of known patterns of cognitive change and their underlying mechanisms. The literature review was used to conceptualize changes in everyday behavior across the spectrum from healthy aging to ADRD and how these changes would be captured by digital phenotyping approaches. For example, the increased variability in standardized cognitive testing and everyday task performance in people with MCI is expected to result in meaningful variability in passive smartphone sensor data in digital phenotyping studies. Without a framework to guide analyses, aggregate data might be prioritized over meaningful variability, which could be misinterpreted as a nuisance (ie, “noise”). Therefore, the VIBE model integrates and extends existing findings to provide structure, guidance, and optimize digital phenotyping study designs.

## Our Proposed Framework

Early stages of pathological aging (ie, MCI) are associated with mild isolated decrements on standardized cognitive tests, subtle difficulties with complex activities of daily living, and increased variability in both cognitive and functional measures. Later stages (ie, dementia) are characterized by greater cognitive and functional impairment, reduced activity and task accomplishment, and less variability in cognitive and functional performance. Table 2 provides a summary of these trends. Multimedia Appendix 1 contains a comprehensive review of the supporting literature [59-107].

**Table 2:** Summary of background literature supporting the Variability in Everyday Behavior (VIBE) framework.

	Healthy aging	Early decline (MCI^a^)	Later decline (dementia)
Cognitive ability	Subtle declines within normative limits	Impaired performance on 1+ domain according to normative scores	Impaired performance on multiple domains according to normative scores
Cognitive variability	Increased variability versus younger adults	Increased variability versus healthy older adults Increased variability predicts further decline and poorer cognition	Less variability than MCI for complex tasks at floor Increased variability than MCI for simple tasks
Everyday functioning	Subtle changes/inefficient behaviors (microerrors) Fully independent	Difficulty with complex tasks Independent with some compensatory strategy use Inefficient (commission errors) and more variable than healthy older adults	Impaired for basic and complex tasks Dependent Outright failure to complete tasks (omission errors)

^a^MCI: mild cognitive impairment.

Theoretical models from computational science offer a useful framework for understanding changes in ability level and variability in the progression of pathological aging. The term “graceful degradation” is used to characterize the way in which complex systems maintain functionality in the face of mild damage or problematic changes in the environment [108]. From a neuropsychological perspective, increased inefficiency and variability in the early stage of decline may stem from faulty executive control mechanisms governed by the prefrontal cortex and associated white matter projections, which, according to a framework proposed by Giovannetti and colleagues [109], are essential to modulate goal activations, enable smooth transitions between goals, and inhibit inappropriate activations from internal or external distractors during everyday tasks. Reductions in extrastriatal dopaminergic neuromodulation required for consistent cognitive control in early stages of dementia support this framework [110-112]. Indeed, long-standing explanations for the link between inconsistency and neurologic disease include impaired neural networks, functional connectivity, and executive functioning [113-115]. An alternative framework from which to interpret early patterns of inefficiency and variability, particularly in the absence of executive function deficits, is the resource theory [116], which originates from the cognitive rehabilitation literature. This theory posits that early damage to any nonspecific brain region depletes overall cognitive resources and leads to errors in task performance and that the level—not the type—of cognitive impairment is critical in determining functioning [109]. As a result of mild resource depletion, compensatory strategies are engaged to allow the system to function, but at a cost (ie, inefficiently, slowly, and inconsistently). In moderate-to-severe stages, greater decrements are observed across multiple cognitive domains, basic activities of daily living are impaired, and patterns of variability are less clear because people are generally less active.

**Figure 1:**
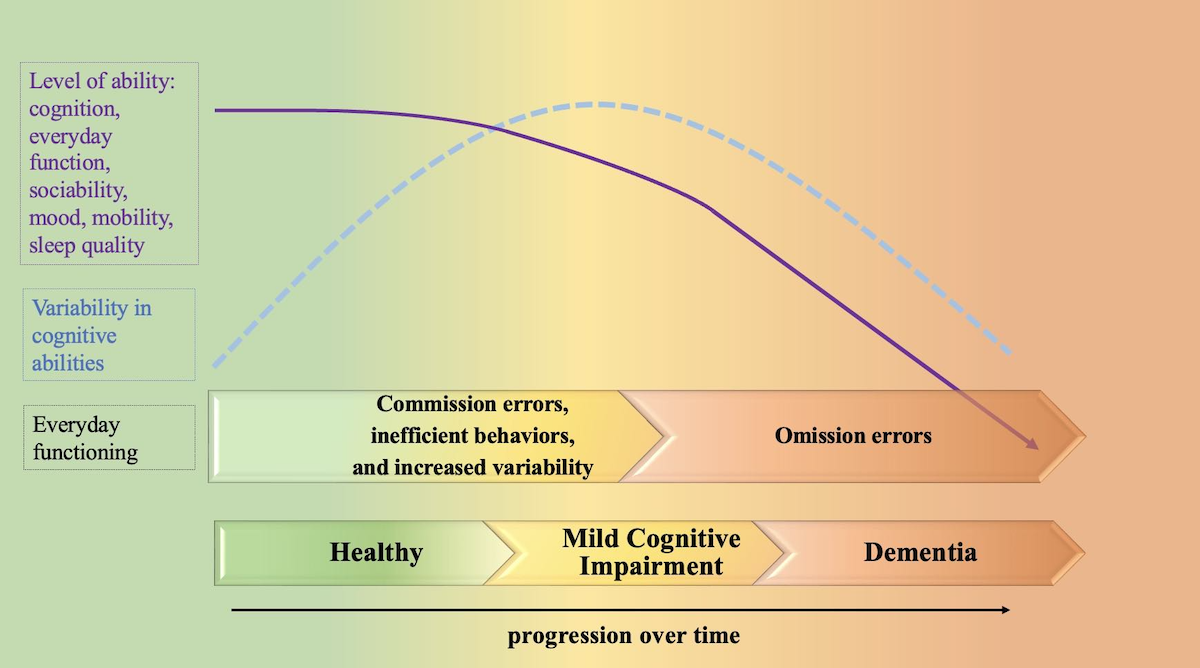
The Variability in Everyday Behavior (VIBE) model of intraindividual variability, cognitive abilities, and everyday functioning for pathological cognitive decline in older adults.

Considering this, we propose the Variability in Everyday Behavior (VIBE) model as a dual-pronged neuropsychological framework that integrates trends in variability (see Figure 1, blue dotted line showing a U-shaped pattern peaking at MCI) and declining ability level (see Figure 1, solid purple line showing a negative linear trend) that are observed across the cognitive aging spectrum. The VIBE model proposes a theoretical foundation from which to evaluate metrics of everyday behavior and cognition captured by the digital phenotyping approach, both in studies examining cross-sectional differences in individuals with different levels of cognitive impairment, and over time in individuals with progressive neurodegenerative disease in longitudinal designs. For example, decreasing cognitive abilities may be indexed by decreases in social activity [117,118], technology usage [119,120], positive mood (ie, increased depressive symptoms [121]), and range of movement/physical activity [122], which can all be inferred from passive sensor metrics. These activity metrics tend to remain stable in earlier stages and begin to decline more notably in the transition from MCI to dementia. A simultaneous examination of intraindividual variability within these metrics across a longitudinal study period may reflect increased day-to-day variability as early as the healthy to MCI transition stage, as individuals engage reserve mechanisms and compensatory strategies to combat mild difficulties and inefficiencies (eg, commission errors). On metrics/activities where dementia-level performance is at floor (eg, movement trajectories outside the home, text messaging, other complex activities where compensatory mechanisms have failed and task goals are no longer achieved; ie, omission errors), we expect variability to decrease below that which we observe in MCI (Figure 1, blue dotted line).

The existing literature is less clear on patterns of variability in the transition from MCI to dementia [123], and we acknowledge the possibility that for relatively simple activities that individuals with mild dementia still perform (eg, movement trajectories within the home, incoming phone calls, sleep/wake cycle), variability may continue to increase in the mild dementia stage followed by eventual decline as abilities further decline. Thus, model predictions should be tested and interpreted with attention to task demands, as well as other contextual features, including the time of day [29], mood, and technology use habits. In other words, the progression from increased variability to decreased variability and complete failure to act depicted in Figure 1 is expected with increasing severity of impairment, though impairment level is determined by more than just clinical status. There may be some period—likely at the transition between MCI and dementia—where contextual factors (eg, task complexity, time of day, external distractors) interact with clinical status to influence level of impairment on metrics of everyday behavior. For example, a person with mild dementia may show marked impairment and decreased variability in financial tasks but may show only mild impairment and increased variability in meal preparation until later in the course of their illness when both tasks are impaired, and variability is diminished. Thus, task effects should be carefully considered, particularly at the boundary of MCI and dementia.

## Application of the VIBE Framework Through Digital Phenotyping Studies

Digital phenotyping using personal smartphone devices represents a promising method to examine age-related changes in functional cognition according to our proposed framework. Study designs may take a variety of forms, but initial studies should include collection of clinically relevant validation measures and longitudinal monitoring. One potential protocol would involve comprehensive baseline assessment to gather gold-standard validation data on function, cognition, mood, and other participant features such as demographics, attitudes toward and experience with technology, and technology use habits that are likely to influence resulting digital data. A period of passive longitudinal monitoring using available, open-source digital phenotyping applications (eg, Beiwe [37], mindLAMP [124]) would involve collection of a host of sensor and application data, including the examples listed in Textbox 1.

The VIBE framework enables systematic selection and analysis of the mobility, sociability, and device activity features from Textbox 1 to obtain activity and variability metrics that could be tested according to a priori hypotheses. A list of nonexhaustive, sample hypotheses derived from the VIBE model that are appropriate for cross-sectional studies of older adults across the cognitive aging spectrum is included in Table 3.

**Example digital phenotyping metrics across 3 feature domains.**

**Mobility**

- Time spent at home
- Distance traveled
- Radius of gyration
- Maximum diameter
- Maximum distance from home
- Number of significant locations
- Average flight length
- Standard deviation of flight length
- Average flight duration
- Standard deviation of flight duration
- Fraction of the day spent stationary
- Significant location entropy
- Minutes of GPS data missing
- Physical circadian rhythm
- Average sleep duration
- Standard deviation of sleep duration

**Sociability**

- Number of outgoing texts
- Total outgoing text length
- Number of incoming texts
- Total incoming text length
- Texting reciprocity
- Texting responsiveness
- Number of outgoing calls
- Total outgoing call duration
- Number of incoming calls
- Total incoming call durations
- Call reciprocity
- Call responsiveness

**Device activity**

- Average battery level
- Total phone off/on events
- Total charge initiations
- Total screen on/off events
- Total application launches
- Application switches
- Central processing unit (CPU) utilization
- Time to view daily survey
- Time to complete daily survey
- Time of first/last screen on event

**Table 3:** Sample hypotheses informed by the Variability in Everyday Behavior (VIBE) model.

Digital phenotyping feature domain	Total activity level metrics	Across-day variability metrics
Mobility	Average distance traveled from home will decline from healthy to MCI^a^ to dementia.	Variability in distance traveled from home will be highest in MCI versus healthy/dementia.
Sociability	Average number of outgoing calls will decline from healthy to MCI to dementia.	Variability in daily average outgoing text length will be highest in MCI versus healthy/dementia.
Device activity	Average number of application launches will decline from healthy to MCI to dementia.	Variability in daily number of screen on/off events will be greater in MCI versus healthy/dementia.
Time of day effects	Average time of first phone use will decline from healthy (earlier) to MCI to dementia (later).	Time of first phone use will be most variable in MCI versus healthy/dementia.

^a^MCI: mild cognitive impairment.

## Methodological Considerations of the Digital Phenotyping Approach

There are a host of important methodological factors that must be thoughtfully considered when conducting such studies, many of which remain unresolved. Cross-device compatibility is a concern that becomes relevant when participants use their own personal devices for data collection. Individual devices may differ in operating system, screen size, sensor sampling rates, and more. These device differences impact user interactions and the quality of data that is collected; they are also related to socioeconomic status and other important participant features and thus cannot be simply covaried in analyses. A single study-issued device may be provided to all participants to standardize data collection and ensure that individuals from underserved groups have an equal opportunity to participate in such studies. However, introducing new technology creates a deviation from participants’ routines, diminishing ecological validity and posing more demands on everyday functioning. Therefore, the personal versus study-provided device decision must be weighed according to the study population and specific aims [27,33]. Although there is a critical concern that studies employing personal digital devices will serve to widen existing health disparities, rates of smartphone ownership—particularly among diverse individuals—have skyrocketed in recent years to include a total of 85% of Americans as of 2021, up from just 35% in 2011 [125]. This rate is consistent across individuals who identify as White (85%), Black (83%), and Hispanic (85%) and is only slightly lower (76%) for individuals with a household annual income less than US $30,000. Therefore, although careful attention must be paid to ensure smartphone studies are equitable, accessible, and generalizable to all, the increased affordability of smartphones may alleviate this concern. Relatedly, recruitment efforts should ensure diverse representation within digital phenotyping studies to investigate the generalizability of these methods. Updates to hardware, software, and allowable permissions (ie, which sensors an app can collect) are occurring at increasingly frequent rates as technology evolves, presenting an additional challenge to the continued validation and generalizability of such approaches. Thus, a device- and operating system–agnostic theoretical model, such as the VIBE model, from which to continually evaluate new data is critically important.

The naturalistic and passive collection of data in a completely unstandardized fashion presents an additional challenge in making between-group comparisons [56], and it remains undetermined whether between-group differences in metrics of interest will emerge despite individual differences in everyday phone use. The most powerful insights from the digital phenotyping approach may be realized by monitoring intraindividual change over longer periods of time, which would require theoretically informed statistical models to make generalizable claims in n-of-1 trials [56]. Another open question relates to the various sampling rates that can be selected to collect raw data from phone sensors and applications, which should be considered in the context of the scientific question and device battery limitations. Although most software platforms include default settings for sensor sampling (eg, GPS sampled at 1 Hz when the phone is in motion, WiFi signals recorded every 5 minutes), they also allow for customization of sampling rates [37]. A variety of GPS sampling rates have been applied across several studies of primarily young adult participants [48,49], and statistical approaches for imputing missing mobility data have been developed [126]. However, limited studies have examined the incremental utility of increased sampling rates across sensors other than mobility for making predictions of interest. Older adult phone users may require less frequent sampling due to less activity, though this may result in a restricted range of variability and impact findings. Determining the minimum necessary sampling frequency for smartphone data is directly tied to feasibility and is critical to inform the design of future studies, as greater frequencies come with greater costs (ie, increasingly expensive sensors, decreased battery life, increased storage needs). This also applies to the optimal length of the data collection period and the study sample size, which may differ depending on the population of interest and the study design [120], and are not appropriately determined using traditional power calculation methods. Barnett and colleagues [127] recommend the use of generalized linear mixed models and change point detection methods to inform the sample size and study duration necessary to achieve adequate power in such studies.

Digital phenotyping studies may employ a combination of passive and active data streams. In active data collection, users are prompted to complete a standardized test or survey on their smartphones, which can be used to yield key contextual information to inform the interpretation of passive sensor data [23,37,128-130]. However, this type of engagement detracts from the unobtrusive, naturalistic nature of pure passive monitoring, and it is unclear which types of active data are most useful when attempting to infer cognition from passive digital data. These methodological questions around sampling frequency and active data collection have not yet been explored in a population of older adult phone users, whose usage patterns may differ and may require increased sampling frequencies or increased active data than younger adults to accurately infer clinically relevant information.

It is also important to establish the context of use of the digital phenotyping approach and determine whether it is best applied as a risk, diagnostic, monitoring, prognostic, or outcome measurement tool. Regulatory agencies like the US Food and Drug Administration and pharmaceutical companies have increasingly recognized the potential of digital devices as a source of “real-world data” and “real-world evidence,” with the capability to monitor health status and clinical response over time and yield new insights about long-term health outcomes in the real world, outside of traditional randomized controlled trials [131]. However, as thoughtfully outlined by O’Bryant and colleagues [9], there are many challenges associated with translating new biomarker discoveries from research domains to routine clinical settings. For this to occur, standardization of the underlying platforms and data frameworks is critical to help make these data more uniform, interoperable, reproducible, and actionable [124]. Questions of scalability, manufacturability, intellectual property law, and regulatory considerations, including inconsistent governance of entities conducting digital health research [132], should not be disregarded [9]. In particular, the point at which mobile digital phenotyping applications are considered “software as a medical device” is ambiguous in the face of rapidly evolving regulatory guidance [133]. Finally, and most importantly, privacy and security concerns must be addressed, and protections of confidentiality must be clearly and continuously communicated to users and participants. Deidentification using study identification numbers, industry-standard encryption methods, storage of encrypted data on secure severs, and ongoing transparency and control over personal data are examples of privacy considerations that should be carefully addressed at the study design phase. Given the extent of personal and sensitive health information involved, prospective risk assessment using tools like the Digital Health Checklist for Researchers should be completed beforehand to evaluate risks and benefits and ensure safe and responsible use of digital tools [132,134]. Importantly, the development and enforcement of privacy standards that are applied consistently across studies will be key to the success of this burgeoning field [35].

## Benefits of the Digital Phenotyping Approach

Despite the numerous unresolved challenges and considerations outlined above, the potential for the digital phenotyping approach to yield ecologically valid and sensitive information on changes in everyday cognition is increasingly apparent. The benefits of emerging digital approaches are outlined in detail in the recent American Psychological Association Handbook of Neuropsychology [57]. To reiterate a few, sample size requirements are reduced when using continuous largescale data, and subtle fluctuations can be captured when data are sampled at such high rates, lending a highly sensitive scale that is captured in vivo. The use of personal smartphone devices represents a complex activity of daily living, thus creating an ideal platform to capture changes that occur early in the disease phase. Early detection of decline provides an opportunity for early intervention, which can lead to notable cost savings and reduced disability-adjusted life years, as noted earlier. Increased smartphone ownership lends increased accessibility compared to traditional methods. Passive data are objective and do not rely on current or retrospective self-report. However, it is possible that the most optimal application of this approach involves a blend of passive phenotyping, ecological momentary assessment for context, and burst cognitive testing to uncover the mechanisms of how changes in cognition within and across days relate to changes in behavior. Additionally, within-person n-of-1 designs may be increasingly sensitive and may address the interpretive challenges of between-groups designs. Finally, emerging digital methods should be considered complementary to traditional neuropsychological evaluations that remain the gold standard tool for diagnosis and intervention. If shown to be valid, emerging digital tools may represent a sensitive and accessible first line measure for early detection, risk stratification, and change in response to interventions.

## Conclusions

Traditional approaches to measuring age-related changes in cognition and function provide valuable and distinct insights. Notable strengths of biomarker, traditional, and mobile cognitive assessments include extensive validation, measurement of discrete cognitive abilities, and localization of pathology (Table 1). At the same time, these approaches present many drawbacks that have become increasingly apparent in the face of technological advances that offer innovative solutions. The digital phenotyping approach is just 1 example of a novel tool that can serve as an increasingly accessible, efficient, sensitive, and personalized complement. Importantly, digital phenotyping remains in its infancy, and many methodological considerations warrant careful attention. Multiple sources of within-person differences (eg, hardware, software, technology habits, daily routines), as well as interpretive challenges of large-scale continuous datasets, make comparisons across individuals and across studies near impossible without a sound theoretical model from which to design and interpret such studies. The VIBE model, supported by decades of cross-discipline literature in neuropsychology, neurology, neuroscience, rehabilitation psychology, and computer science, proposes testable hypotheses (see Figure 1 and Table 3) that can be used in future digital phenotyping studies to provide novel, valuable, and clinically interpretable insights into meaningful changes in everyday behavior and cognition.

## Appendix

Multimedia Appendix 1 Supporting background literature.

## Abbreviations

AD: Alzheimer’s disease
ADRD: Alzheimer’s disease and related dementias
CSF: cerebrospinal fluid
MCI: mild cognitive impairment
NIA: National Institute on Aging
NIH: National Institutes of Health
VIBE: Variability in Everyday Behavior
