# Supplementary material for: Capturing Cognitive Aging in Vivo: Application of a Neuropsychological Framework for Emerging Digital Tools
Source: JMIR Aging. 2022 Sep 7;5(3):e38130. doi: 10.2196/38130 (PMC9494215; doi:10.2196/38130)
Supplement: Multimedia Appendix 1 [file aging_v5i3e38130_app1.docx]

## Known Trends in Cognitive and Functional Abilities

### Healthy Aging

Decades of neuropsychological research documents predictable trends in cognition and function along the pathological aging spectrum (normal cognition to prodromal MCI to a dementia diagnosis). Over the course of healthy aging, subtle declines across cognitive domains including processing speed, executive functioning, working memory, and free recall are expected and correspond to subtle changes in everyday functioning [86]. Older adults also exhibit increased variability in cognitive test scores as compared to younger adults, performing less consistently on a given task across testing visits, across domains within a single visit, and across trials within a single task [74,87]. Higher levels of intraindividual variability have also associated with poorer scores on complex cognitive tasks among healthy older adults [88]. This pattern of increased variability relative to younger adults, along with mild declines in cognitive abilities and everyday function, is expected and corresponds to normative age-related changes in brain structure and function [89,90].

### Early-Stage Patterns of Decline (MCI)

#### i. Level of cognitive ability

On standardized neuropsychological testing, individuals with MCI perform 1-1.5 standard deviations below the demographically-adjusted mean on one to two tests within a single domain, or on one test within multiple domains [7,16,91,92]. Recent research criteria have also proposed increasingly sensitive cut-offs for a pre-MCI stage termed subtle cognitive decline [93]. Regardless of the criteria applied, individuals with underlying neurodegenerative pathology show decrements on standardized norm-adjusted neuropsychological test scores that differentiate them from cognitively typical older adults.

#### ii. Variability in cognitive performance

Qualitatively, there is notable variability and inefficiency in cognitive task performance, as reserve mechanisms are recruited to compensate for these early, mild difficulties. Increased levels of intraindividual variability on tests of cognition are related to poorer cognitive performance overall, are elevated in MCI as compared to healthy older adults, and predict further decline [87,94–96]. In one longitudinal study of older adults aged 54-89 years, MacDonald and colleagues [97] found that increased across-trial reaction time intraindividual variability at baseline was associated with worsening performance over a six-year period across a variety of cognitive abilities, including perceptual speed, working memory, and fluid reasoning. Lövdén and colleagues [94] extended this work using a structural equation model approach to show that increases in trial-to-trial reaction time variability do in fact precede changes in overall performance, further indicating that increases in variability represent an early sign of impending decline. Other studies have identified similar predictive utility in variability metrics aside from reaction time variability. Single-session across-task variability (or dispersion) has been associated with subsequent cognitive decline in community-dwelling older adults [74,98] and has been shown to predict conversion from MCI to AD [64,99,100]. In another study among individuals with MCI, greater baseline dispersion was associated with lower entorhinal cortical thickness and poorer functioning cross-sectionally, along with greater reduction in entorhinal thickness, hippocampal volume, and functioning over time [101], suggesting intraindividual variability is a sensitive marker of neurodegeneration and functional decline.

#### iii. Everyday functioning

In parallel to declining cognitive ability and increased variability on standardized cognitive tasks, similar changes are observed in everyday activities during early stages of decline. On activities of daily living, individuals with MCI remain “functionally independent” according to diagnostic criteria, yet experience difficulty with complex tasks (i.e., financial and medication management, meal preparation, appointment management, technology use; as demonstrated across various studies [102–106]). In everyday life, they are less efficient, take longer to complete certain tasks, have difficulty sustaining effort or inhibiting distractions, and rely increasingly on compensatory strategies. Further, those with MCI who experience greater difficulties with everyday tasks are at greater risk to progress to dementia [107,108].

Increased inefficiency and variability in everyday functioning also emerge on standardized, performance-based measures of functional abilities. In a study examining performance on a standardized everyday task (Naturalistic Action Task; NAT), Giovannetti and colleagues compared error profiles across healthy controls, individuals with MCI, and those with AD [109] and found differences in the number and type of errors across the groups. People with MCI made an intermediate number of errors compared to controls and people with dementia. Additionally, people with MCI made a greater number of commission errors- the inaccurate performance of a task step due to mis-sequencing or use of an inappropriate tool/object - as compared to normal controls, even though they accomplished the same number of task steps. Relative to people with AD, individuals with MCI accomplished significantly more task steps. Commission errors made by people with MCI were conceptualized as errors of efficiency or executive control over intact schema for task goals, because essential task steps were ultimately performed, albeit inaccurately/inefficiently [109].

Other studies employing in-home motion detection sensors identified variability metrics that differentiated healthy older adults from those with MCI. In a study by Dodge and colleagues [110], unobtrusive sensors assessed walking speed trajectories of older adults with normal cognition, non-amnestic MCI and amnestic MCI during a period of over two years. In examining walking speed variability (i.e., coefficient of variability; COV), investigators found that individuals with non-amnestic MCI demonstrated a distinct trajectory of variability, characterized by the greatest walking speed variability at baseline followed by a further increase in variability over time. Numerous other studies have identified associations between variability in gait speed or movement trajectories and MCI status or conversion to AD [111–116]. Potential mechanisms for gait variability as a marker of early cognitive decline include the fact that slowing gait has been related to periventricular white matter changes [117,118], total and periventricular white matter hyperintensity burden [119], as well as a number of other neuropathologies [110], suggesting a multifactorial relation between gait variability and cognition.

Studies instrumenting at-home objects with passive sensors have also identified similar patterns of increased inefficiency and variability in everyday activities. Investigators using an in-home sensor-based assessment platform from the Oregon Center for Aging and Technology have observed patterns in variability across a variety of ecologically valid, in-home behaviors. These include day-to-day computer use, which showed lower frequency and increasing variability over time in those with MCI as compared to normal controls [70]; higher variability in time to take medications at baseline and over time in older adults with poorer cognition [120]; and more variable, less efficient computer mouse movements in those with MCI versus those with normal cognition [121].

### Later-Stage Patterns of Decline (Dementia)

#### i. Level of cognitive ability

Later stages of decline – when individuals progress from MCI to frank dementia – are characterized by greater severity of both cognitive and functional impairment. According to the latest published diagnostic criteria [10], syndromal staging of dementia is characterized by substantial progressive cognitive impairment with globally impaired scores across multiple versus isolated cognitive domains. The central feature that differentiates dementia from MCI is the evident functional impact on daily life; persons with dementia are no longer independent and require assistance with activities of daily living.

#### ii. Variability in cognitive performance

Patterns of variability on cognitive testing are less conclusive in later stages of decline as compared to the consistent uptick in variability observed in MCI. In a study by Reckess and colleagues [122], the within-person distribution properties on a cognitive test battery was examined across a range of older adults from healthy to those with progressively more severe cognitive impairment. They observed that across-test intraindividual variability– or test score dispersion – expands in MCI but shrinks in dementia. They interpreted these findings from a psychometric standpoint, noting that the neurodegenerative disease pervading most domains of cognition causes a shift towards lower scores and a contraction of the test-score dispersion [122]. In other words, when neuropsychological performance is already near floor, variability will tend to decrease. On the other hand, in a separate study of a total of 819 AD, MCI and healthy participants from the Alzheimer’s Disease Neuroimaging Initiative database, intraindividual variability across cognitive tasks showed a stepwise increase from healthy to MCI to AD, with the AD group showing a significantly higher baseline intraindividual variability as compared to the MCI group [100]. Other studies have found higher intraindividual variability in those with mild dementia as compared to healthy controls, focusing on variability in latency on reaction-time tasks both across-trials and across-occasions [123]. Generally, greater intraindividual variability tends to be associated with the severity of neurological dysfunction [87], though studies directly comparing MCI and AD groups are limited and results may depend on the type of variability metric used as well as the difficulty of the cognitive tasks concerned.

#### iii. Everyday functioning

On everyday tasks, individuals with mild-moderate dementia show marked impairment on not just complex tasks but also basic activities of daily living and are no longer able to function independently. Difficulties with everyday activities are common and lead to numerous negative health outcomes, including quality of life [124], depression [125], institutionalization [126], death [127], and caregiver burden [128]. On standardized everyday tasks, individuals with dementia make a greater number of total errors than people with MCI or normal cognition [129]. People with dementia also make more omission errors - where task steps are left out completely. This trend has been observed across a variety of studies comparing individuals with dementia to those with schizophrenia [130], healthy older adults [109,131], and those with MCI [109,132]. Omission errors made by people with dementia are conceptualized as degraded knowledge of everyday tasks (i.e., degraded representation of task schema or goals [60]).

The overt decline in everyday functioning among persons with dementia has also been captured in studies employing passive and naturalistic digital measures, though notably fewer studies have examined participant groups at more severe stages of decline. One such study using passive infrared sensors installed in participants’ homes found that individuals with dementia-level cognitive impairment had significantly fewer outings than healthy controls [133]. A similar pattern of reduced mobility in dementia as compared to people with MCI and healthy cognition was shown in a study by Mattek and colleagues [134] using wearable wrist-worn device data over a period of 10 months.
